# Correction: Women’s household decision-making power and contraceptive use in Mali

**DOI:** 10.1186/s12978-023-01569-0

**Published:** 2023-03-22

**Authors:** Abdul‑Aziz Seidu, Bright Opoku Ahinkorah, Ebenezer Kwesi Armah‑Ansah, Louis Kobina Dadzie, Richard Gyan Aboagye, Edward Kwabena Ameyaw, Eugene Budu, Betregiorgis Zegeye, Sanni Yaya

**Affiliations:** 1 Department of Population and Health, University of Cape Coast, Cape Coast, Ghana; 2 College of Public Health, Medical and Veterinary Services, James Cook University, Townsville, Australia; 3 Department of Estate Management, Takoradi Technical University, Takoradi, Ghana; 4 School of Public Health, Faculty of Health, University of Technology Sydney, Sydney, Australia; 5 L & E Research Consult Limited, Wa, Ghana; 6 School of Public Health, University of Health and Allied Sciences, Ho, Ghana; 7 HaSET Maternal and Child Health Research Program, Shewarobit Field Office, Shewarobit, Ethiopia; 8 School of International Development and Global Studies, Faculty of Social Sciences, University of Ottawa, 120 University Private, Ottawa, ON K1N 6N5 Canada; 9 The George Institute for Global Health, Imperial College London, London, UK

**Correction: Reproductive Health (2022) 19:232** 10.1186/s12978-022-01534-3

After publication of this article [1], the authors made a few edits to the the text and tables. The changes are shown in Additional file 1.

The original article [1] has been corrected.

## Supplementary Information

**Additional file 1.** Original article with detailed corrections.
