# Supplementary material for: Correction: Women’s household decision-making power and contraceptive use in Mali
Source: Reprod Health. 2023 Mar 22;20:48. doi: 10.1186/s12978-023-01569-0 (PMC10035172; doi:10.1186/s12978-023-01569-0)
Supplement: Supplementary file 1 — Additional file 1. Original article with detailed corrections. [file 12978_2023_1569_MOESM1_ESM.pdf]

Reproductive Health

RESEARCH

Open Access

Women's household decision-making power and contraceptive use in Mali

Abdul-Aziz Seidu[1,2,3], Bright Opoku Ahinkorah[4], Ebenezer Kwesi Armah-Ansah[1], Louis Kobina Dadzie[1*], Richard Gyan Aboagye[6], Edward Kwabena Ameyaw[4,5], Eugene Budu[1], Betregiorgis Zegeye[7] and Sanni Yaya[8,9]

Abstract

Background: Utilization of contraceptives remains low in several countries in sub-Saharan Africa despite evidence of its benefits. Several factors are associated with contraceptive use. However, little is known about the association between women's decision-making capacity and the utilization of contraceptives in Mali. This study sought to determine the ~~effect of~~ women's household decision-making power ~~on~~ contraceptive use in Mali.

Methods: This study involved a cross-sectional analysis of data from the 2018 Mali Demographic and Health Survey. A total of 7893 married women were included in the final analysis. A binary logistic regression analysis was conducted with statistical significance set at $p < 0.05$.

Results: Contraceptive use among married women in Mali was 17.1%. The odds of using contraceptives were higher among women with joint decision-making with their husbands on how to spend respondent's earnings [aOR = 1.79; 95% CI = 1.12, 2.85], joint decision-making with their husbands on what to do with their husband's earnings [aOR = 1.43; 95% CI = 1.12, 1.83], and joint decision-making with ~~husband~~ on large household purchases [aOR = 1.32; 95% CI = 1.10, 1.59]. Deciding alone on a visit to family or relatives was associated with lower odds of contraceptive use [~~AOR~~ = 0.72; 95% CI = 0.58, 0.89].

Conclusion: The study has revealed that joint household decision-making is positively associated with contraceptive use. Therefore, to achieve Sustainable Development Goal 3, the ministry for the advancement of women, children and families and related stakeholders must unearth strategies to empower women in joint decision-making and encourage men's involvement in contraceptive decision-making.

Keywords: Contraceptive use, Household decision-making power, Mali, Demographic and Health Survey

Introduction

The population of sub-Saharan Africa (SSA) is expected to double by the next four decades [1]. This has made the ever-increasing population growth a major concern in SSA since the fertility rate ranges from 2.9 in Botswana to 7.2 in Niger [2]. The variations in fertility rates across the countries in SSA as a result of low utilization of contraceptives and high unmet needs for contraceptive [3, 4]. This has made contraceptive use a critical tool due to weakened maternal and child health care services [5]. The use of contraceptives contributes positively to the socioeconomic and health outcomes of couples and their families [6]. That ~~is~~, contraceptive allows women, men and couples to either voluntarily or otherwise decide on birth delaying, spacing and child limiting [7]. Studies show that contraceptive use has at least reduced pregnancies and unsafe abortions whilst increasing birth spacing and pregnancy delay prospects [5, 8].

Despite considerable investments and funding of contraceptive programs, usage is still low in most countries

*Correspondence: louis.dadzie1@stu.ucc.edu.gh

[1] Department of Population and Health, University of Cape Coast, Cape Coast, Ghana
Full list of author information is available at the end of the article

© The Author(s) 2022. **Open Access** This article is licensed under a Creative Commons Attribution 4.0 International License, which permits use, sharing, adaptation, distribution and reproduction in any medium or format, as long as you give appropriate credit to the original author(s) and the source, provide a link to the Creative Commons licence, and indicate if changes were made. The images or other third party material in this article are included in the article's Creative Commons licence, unless indicated otherwise in a credit line to the material. If material is not included in the article's Creative Commons licence and your intended use is not permitted by statutory regulation or exceeds the permitted use, you will need to obtain permission directly from the copyright holder. To view a copy of this licence, visit http://creativecommons.org/licenses/by/4.0/. The Creative Commons Public Domain Dedication waiver (http://creativecommons.org/publicdomain/zero/1.0/) applies to the data made available in this article, unless otherwise stated in a credit line to the data.

in SSA [5, 9]. However, in 2017, more than two-thirds of married women in their reproductive ages and their partners used any modern or traditional methods of contraceptives [10]. It is believed that about 225 million people in low-and-middle-income countries who are in their reproductive ages are not using any method of contraceptive [11]. Evidence shows that socio-economic and demographic factors including place of residence, household head, educational level, age, marital status, religion and other factors have been linked to the utilization of contraceptives in SSA [5]. The varied nature of SSA countries has affected their sexual and reproductive health outcomes leading to a considerably high number of unsafe abortions [12, 13]. It is on record that most women in low-and-middle-income countries (LMICs) have an unmet need for contraceptives [14, 15].

Mali, one of the countries in SSA, is the third highest in the world with a fertility rate of 6.3 compared with 7.1 births per woman about three decades [16, 17]. The population of Mali which is around 20 million is estimated to double in the next one and a half decade [16]. This has been attributed to the estimated low prevalence of modern contraceptive utilization and their traditional practice of early marriage which has initiated a lot of females to early childbearing [17, 18]. Contraceptive use remains far from ideal as current estimates reveal that less than a quarter of married adolescents and married women in their reproductive age use modern contraceptives [17].

This has compelled the government of Mali and development partners to initiate programs that will reposition contraceptives as an essential public health and development intervention [17]. These initiatives have subsequently led to cost reduction of contraceptives, and made contraceptives more accessible with strengthened reproductive health education of religious leaders and parliamentarians [19]. However, these interventions are not likely to see the light of day due to the traditional norms which uphold high fertility and gender inequality that restricts women's ability to make decisions in contraceptive [20]. That is, the sociocultural variations which are still in force have deprived women of contraceptive usage [2].

Although access to economic resources, the political, social and health status of women have seen some improvement, globally, women's empowerment is essential in contraceptive utilization [5, 21]. Empirical evidence reveals that household decision-making does not favour women unlike their men counterpart [22]. It has also been noted that the modernization of many subcultures which has allowed women to receive higher education, engagement in the labour force, marry at an older age, choose their partner and live apart from their extended families have been associated with a greater women's household decision-making [23].

A couple of researches on SSA have revealed that the status of women at the household level hurts their reproductive desires or contraceptive use [24]. In SSA, women's low self-esteem status at the household level has placed them at a weaker level which has negatively undermined their achievement of desired reproductive and contraceptive use because men or their spouses are the decision-making authority [24–27]. The patriarchal ideology in most SSA societies, including Mali, has often seen women relegated to the backstage has led to unplanned pregnancies and unsafe abortion [28, 29].

Also, there is an impressive body of literature that has examined the role of decision-making power in contraceptive use and inadequate resource-constraint settings [23]. Some studies have shown that women's household decision-making influences their ability to make decisions about contraceptive use [5, 21, 30]. However, studies have not focused on the role of women's household decision-making power and contraceptive use in Mali. Hence, we examined the association between the household decision-making power of women and contraceptives in Mali. This study aside from addressing the gap in literature will not only provide the basis for policymakers to design appropriate policies and programs but will make recommendations that will help increase contraceptive use in Mali.

Methodology

Data source and sample design

This study involved a cross-sectional analysis of data from the 2018 Mali Demographic and Health Survey (MDHS). The data for the study was specifically extracted from the women's file. The MDHS forms part of the Demographic and Health Survey (DHS) program which aims at monitoring health indicators in low and middle-income countries [31]. The DHS collects data on several health and social issues such as the use of contraceptives [31]. DHS employed a multistage cluster sampling technique in recruiting respondents for the study. In the first stage, 379 Primary Survey Units (UPS) or clusters (104 in urban and 275 in rural areas) were systematically drawn with a probability proportional to their size in households, from the list of Enumeration Sections (SE). A total of 35 households each in the Kidal, Gao, and Timbuktu regions respectively and 26 households in all the other regions with a systematic draw with equal probability were sampled. In the selected households, all women aged 15–49 usually living in the households, or present the night before the survey, were eligible to be surveyed. For this study, a total of 7893 currently married women with complete cases of variables of interest were

included in the analysis. We relied on the Strengthening the Reporting in Observational Studies in drafting this manuscript [32].

Study variables

Dependent variable

The dependent variable for the study was contraceptive use. This was defined as the percentage of married women aged 15–49 years who are currently using contraceptives. If a woman used any of the following she was considered to be using contraceptives; pills, IUDs, injectables, condoms, sterilisation, implants/Norplant, periodic abstinence and withdrawal [33]. The outcome was in a binary form coded as '1' = using modern contraception and '0' = non-use of modern contraception.

Independent variables

Women's autonomy which incorporated women's control over their earnings and that of their husbands, the decision on their healthcare, decision on purchasing large household items and decisions on visiting family/relatives formed the main independent variables. These variables were recoded into "women only', 'joint with husband' and 'husband only' as decision makers to indicate the level of involvement in decision-making in the household [33]. Other socio-demographic and economic variables such as age, residence, number of living children, educational level, work status and wealth were included in the study. Educational level was recoded as 'no education', 'primary' and 'secondary/higher'. Work status was also recoded as 'not working', 'sales', 'agricultural', and others. The number of living children was recoded from '0' to '5 + '. Conversely, age, place of residence and wealth status remained unchanged.

Data analysis

Bivariate analysis was first conducted and the significant levels were examined using the Chi-square test. A binary logistic regression was employed to determine the effect of each of the main independent variables (control over earnings, decision on healthcare, decision on purchasing of large household items and decision on visiting family or relatives) on the dependent variable. On the other hand, adjustments for the other independent variables (age, educational level, number of living children, residence, occupation and wealth) were made separately for the main independent variables on the outcome. Crude and adjusted odds ratios were calculated from the binary and multiple logistic regressions. We made use of the weighting factor to adjust for over and under-sampling in the dataset. Stata version 14.0 was used for the analysis. Significant levels were set at $p < 0.05$ with the associated confidence intervals. Results were presented in tables.

Results

Contraceptive prevalence rate (CPR) among currently married women in the reproductive age groups

We found that 17.1% of currently married women within the reproductive age were contraceptive users. The highest proportion of women who used contraceptives (28.8%) made decisions on respondents' earnings jointly with their husbands. With regards to a decision on husbands/partners' earnings, about 17% of the respondents indicated husbands only. Among contraceptive users, 21%, 22.9% and 22.3% made joint decisions with husbands on respondents' healthcare, large household purchases and decision on visits to family or relatives respectively. A little over 20% of the respondents who used contraceptives were aged 35–39 yrs. The highest proportion (20.1%) of those using contraception had five or more living children. Again, 22.1% of the urban dwellers were contraceptive users. Moreover, close to 30% of respondents with secondary/higher educational levels were contraception users for both women and husbands/partners. Contraceptive prevalence was highest (24.3%) among the richest wealth quintile and lowest (11.5%) among the poorest (see Table 1).

Decision-making variables and their association with contraceptive use in Mali

The odds of using contraceptives were higher among women with joint decision-making with their husbands on how to spend respondent's earnings [aOR = 1.79; 95% CI = 1.12, 2.85], joint decision-making with their husbands on what to do with their husband's earnings [aOR = 1.43; 95% CI = 1.12, 1.83], and joint decision-making with husband on large household purchases [aOR = 1.32; 95% CI = 1.10, 1.59]. Deciding alone on a visit to family or relatives was associated with lower odds of contraceptive use [AOR = 0.72; 95% CI = 0.58, 0.89] (Table 2).

Discussion

This paper examined the association between women's household decision-making power and contraceptive use in Mali. This study found a low prevalence rate (17.1%) of contraceptive use among currently married women aged 15–49 years. The prevalence of rate of this is study's findings are supported by a similar study in a study in Mali [34]. Studies have identified the practice of a patriarchal system in Malian society as a key contributor to the low contraceptive prevalence rate in the country [35]. Malian society is mainly patriarchal in the sense that culture delegates authority to men hence currently married women may perhaps have little autonomy over their bodies, mobility, and finances which may affect their accessibility to contraceptive services. In such a cultural domain,

Table 1 Contraceptive prevalence rate (CPR) among currently married women in the reproductive age groups by selected variables

Variable	Contraceptive usage			
	Not using (82.9%)		Using (17.1%)	
	Percent	95% CI	Percent	95% CI
Decides how to spend respondent's earnings*				
Women only (n = 2661)	80	[77.7–82.1]	20	[17.9–22.3]
Joint with husband (n = 198)	71.2	[63.8–77.6]	28.8	[22.4–36.2]
Husband only (n = 431) ‡	81.4	[76.5–85.5]	18.6	[14.5–23.5]
Decides what to do with husband's earnings**				
Women only (n = 498)	82	[77.1–86.1]	18	[13.9–22.9]
Joint with husband (n = 568)	76	[71.0–80.5]	24	[19.5–29.0]
Husband only (n = 7135)‡	83.4	[81.9–84.9]	16.6	[15.1–18.1]
Decides on respondent's health care*				
Women only (n = 588)	84	[79.9–87.4]	16	[12.6–20.1]
Joint with husband (n = 1120)	79	[75.3–82.3]	21	[17.7–24.7]
Husband only (n = 6542)‡	83.3	[81.8–84.8]	16.7	[15.2–18.2]
Decides on large household purchases***				
Women only (n = 518)	82	[77.4–85.9]	18	[14.1–22.6]
Joint with husband (n = 1248)	77.1	[73.6–80.3]	22.9	[19.7–26.4]
Husband only (n = 6484)‡	83.9	[82.4–85.3]	16.1	[14.7–17.6]
Decides on visits to family or relatives**				
Women only (n = 1044)	85.7	[82.6–88.3]	14.3	[11.7–17.4]
Joint with husband (n = 1296)	77.7	[74.0–81.1]	22.3	[18.9–26.0]
Husband only (n = 5910)‡	83.3	[81.6–84.9]	16.7	[15.1–18.4]
Age in 5-year groups***				
15–19 (n = 871)	90.6	[88.0–92.6]	9.4	[7.4–12.0]
20–24 (n = 1509)	82.8	[80.1–85.2]	17.2	[14.8–19.9]
25–29 (n = 1778)	81.5	[79.0–83.7]	18.5	[16.3–21.0]
30–34 (n = 1480)	80.5	[77.6–83.1]	19.5	[16.9–22.4]
35–39 (n = 1233)	78.9	[75.7–81.8]	21.1	[18.2–24.3]
40–44 (n = 822)	83.2	[79.9–86.1]	16.8	[13.9–20.1]
45–49 (n = 557)	90.7	[87.4–93.2]	9.3	[6.8–12.6]
Number of living children***				
0 (n = 767)	97.7	[96.1–98.7]	2.3	[1.3–3.9]
1 (n = 1247)	85.5	[82.9–87.8]	14.5	[12.2–17.1]
2 (n = 1349)	81.6	[78.8–84.1]	18.4	[15.9–21.2]
3 (n = 1256)	81.9	[79.3–84.3]	18.1	[15.7–20.7]
4 (n = 1175)	80.4	[77.3–83.2]	19.6	[16.8–22.7]
5 + (n = 2456)	79.9	[77.5–82.2]	20.1	[17.8–22.5]
Place of residence***				
Urban (n = 2341)	77.9	[74.9–80.7]	22.1	[19.3–25.1]
Rural (n = 5909)	84.3	[82.5–85.9]	15.7	[14.1–17.5]
~~Highest educational~~ level of respondent***				
No education (n = 6026)	85.7	[84.0–87.2]	14.3	[12.8–16.0]
Primary (n = 1005)	81.5	[78.3–84.3]	18.5	[15.7–21.7]
Secondary/Higher (n = 1219)	70.5	[67.1–73.6]	29.5	[26.4–32.9]
Respondent's occupation***				
Not working (n = 3781)	86.3	[84.6–87.8]	13.7	[12.2–15.4]
Sales (n = 1933)	77	[74.4–79.4]	23	[20.6–25.6]
Agricultural sector (n = 1761)	84.5	[81.0–87.4]	15.5	[12.6–19.0]
Others (n = 775)	79.2	[75.3–82.6]	20.8	[17.4–24.7]

Table 1 (continued)

Variable	Contraceptive usage			
	Not using (82.9%)		Using (17.1%)	
	Percent	95% CI	Percent	95% CI
Husband/partner's education level***				
No education (n = 6200)	85.6	[83.9–87.1]	14.4	[12.9–16.1]
Primary (n = 729)	80.7	[77.4–83.7]	19.3	[16.3–22.6]
Secondary/Higher (n = 1321)	71.5	[68.6–74.3]	28.5	[25.7–31.4]
Wealth index***				
Poorest (n = 1533)	88.5	[85.5–90.9]	11.5	[9.1–14.5]
Poorer (n = 1575)	88.3	[85.7–90.5]	11.7	[9.5–14.3]
Middle (n = 1677)	82.9	[80.3–85.3]	17.1	[14.7–19.7]
Richer (n = 1796)	79.3	[76.4–81.9]	20.7	[18.1–23.6]
Richest (n = 1669)	75.7	[72.7–78.4]	24.3	[21.6–27.3]

Missing values are excluded from the calculations

‡ Including a small percentage of others

***p < 0.001, **p < 0.01, *p < 0.05

Table 2 Decision-making variables and their association with contraceptive use in Mali

Variable	COR	AOR[a]
Decides how to spend respondent's earnings		
Women	1.09 [0.81,1.47]	1.06 [0.77,1.45]
Joint with husband	1.77**[1.15,2.74]	1.79* [1.12,2.85]
Husband *(ref)*	1 [1.00,1.00]	1 [1.00,1.00]
Decides what to do with husband's earnings		
Women	1.1 [0.84,1.45]	1.01 [0.76,1.33]
Joint with husband	1.59*** [1.26,2.00]	1.43** [1.12,1.83]
Husband *(ref)*	1 [1.00,1.00]	1 [1.00,1.00]
Decides on respondent's health care		
Women	0.96 [0.74,1.23]	0.9 [0.69,1.16]
Joint with husband	1.33** [1.11,1.60]	1.15 [0.94,1.39]
Husband (ref)	1 [1.00,1.00]	1 [1.00,1.00]
Decides on large household purchases		
Women	1.14 [0.88,1.48]	1.04 [0.79,1.38]
Joint with husband	1.55*** [1.30,1.84]	1.32** [1.10,1.59]
Husband *(ref)*	1 [1.00,1.00]	1 [1.00,1.00]
Decides on visits to family or relatives		
Women	0.83 [0.68,1.02]	0.72** [0.58,0.89]
Joint with husband	1.43*** [1.20,1.70]	1.19 [0.98,1.43]
Husband *(ref)*	1 [1.00,1.00]	1 [1.00,1.00]

[a] Adjusted for age, number of living children, residence, respondents' educational level, work, husbands' educational level and wealth

***p < 0.001, **p < 0.01, *p < 0.05

females obtaining contraception is often very difficult and looked down upon.

After adjustment for confounders, our final model highlights a total of four decision-making variables which showed significant association with contraceptive use among currently married women. These key decision-making variables are the decision on how to spend respondent's earnings, what to do with husband's earnings, decision on large household purchases and decision on visits to family or relatives.

The strongest independent determinant of contraceptive use among the study population was joint decision-making. Regarding economic decisions, women who take joint decisions with their husbands on how to spend their earnings and what to do with their husband's earnings have higher odds of using contraceptives compared to women whose husbands entirely decide on these economic issues. Our findings concur with evidence derived from earlier studies in Nigeria and Bangladesh [36, 37]. Blackstone and Iwelunmor [36] found a lower likelihood of modern contraceptive use among couples in which male partners hold primary decision-making power in Nigeria. Our findings also affirm what Uddin et al. [37] found in Bangladesh. They found that, compared to a spouse who jointly makes decisions about major household purchases, the odds of using contraceptives was 24% lower (OR 0.76; 95% CI 0.55- 1.05) among households where the husband is the sole decision maker about major household purchases. The possible explanation might be that women who can take part in household decision-making were also able to share in decisions associated to contraceptive use. Participation in decision-making power on contraceptive use are more related to women's autonomy to decide jointly with their husband on issues relating to their reproductive life.

The findings also revealed a significant association between the decision on visits to family or relatives. Uddin et al. [37] found that the involvement of the husband alone or others in decisions particularly concerning the visit to family or relatives is associated with lower contraceptive use compared to concordant joint decision-making in Bangladesh. The plausible explanation was that when women's freedom to physical mobility is usually controlled by husbands or other family members, it may restrict their access to reproductive health resources and information. The authors attribute the authority to control women's physical mobility to the Muslim institution of purdah in Bangladesh. In contrast, we found that the involvement of women alone in decisions concerning visits to family or relatives is significantly associated with lower contraceptive use compared to decision-making by the husband alone. This finding can plausibly be explained by the argument that not all decision-making items are strategically important for women's autonomy and power as far as decisions on contraceptive use are concerned.

Strengths and limitations

Since these findings are cross-sectional, we were limited in our ability to infer causation and can only demonstrate associations between contraceptive use and potential indicators of decision-making power. Regardless of these limitations, the strength of the study lies in the relatively large sample size that gave the study the statistical power to run rigorous analyses. Also, our findings are generally consistent with existing findings.

Conclusion

The results of the study showed that joint household decision-making is a good predictor of married women's contraceptive use in Mali. Contrarily, women's decision alone about visits to family and relatives was found to be less likely to predict contraceptive use. As a result, it is recommended to the ministry for the advancement of women, children and families and related stakeholders to improve efforts to empower women in joint decision-making and encourage men's involvement in contraceptive decision-making. These could help in the attainment of Sustainable Development Goal 3 and the decline in the alarming population growth.

Acknowledgements
Not applicable.

Author contributions
AS and BOA conceptualized the study; AS designed the study; LKD, AS, BOA, and EKA carried out the analysis; LKD, RGA, EB, EKA, BOA and AS interpreted the results; EKA-A, BZ, SY, EB and RGA assisted in drafting and reviewing the manuscript; AS, BOA and EKA supervised the course of the article. All authors read and approved the final manuscript.

Funding
No funding.

Availability of data and materials
The datasets generated and/or analysed during the current study are available in the DHS program repository, https://dhsprogram.com/data/dataset.

Declarations

Ethics approval and consent to participants
Not applicable.

Consent for publication
Not applicable.

Competing interests
The authors declare that they have no competing interests.

Author details
[1] Department of Population and Health, University of Cape Coast, Cape Coast, Ghana. [2] College of Public Health, Medical and Veterinary Services, James Cook University, Townsville, Australia. [3] Department of Estate Management, Takoradi Technical University, Takoradi, Ghana. [4] School of Public Health, Faculty of Health, University of Technology Sydney, Sydney, Australia. [5] L & E Research Consult Limited, Wa, Ghana. [6] School of Public Health, University of Health and Allied Sciences, Ho, Ghana. [7] HaSET Maternal and Child Health Research Program, Shewarobit Field Office, Shewarobit, Ethiopia. [8] School of International Development and Global Studies, Faculty of Social Sciences, University of Ottawa, 120 University Private, Ottawa, ON K1N 6N5, Canada. [9] The George Institute for Global Health, Imperial College London, London, UK.

Received: 26 September 2022 Accepted: 22 November 2022
Published online: 28 December 2022

References

1. O'Regan A, Thompson G. Indicators of young women's modern contraceptive use in Burkina Faso and Mali from Demographic and Health Survey data. Contracept Reprod Med. 2017;2(1):1–8.
2. Adedini SA, Omisakin OA, Somefun OD. Trends, patterns and determinants of long-acting reversible methods of contraception among women in sub-Saharan Africa. PLoS ONE. 2019;14(6): e0217574.
3. Imasiku EN, Odimegwu CO, Adedini SA, Ononokpono DN. Variations in unmet need for contraception in Zambia: does ethnicity play a role? J Biosoc Sci. 2014;46(3):294–315.
4. Bongaarts J. Africa's unique fertility transition. Popul Dev Rev. 2017;1(43):39–58.
5. Islam AZ. Factors affecting modern contraceptive use among fecund young women in Bangladesh: does couples' joint participation in household decision making matter? Reprod Health. 2018;15(1):1–9.
6. Belohlav K, Karra M. Household decisionmaking and contraceptive use in Zambia. Washington: Population Reference Bureau; 2013.
7. Namasivayam A, Lovell S, Namutamba S, Schluter PJ. Improved contraceptive use among women and men in Uganda between 1995–2016: a repeated cross-sectional population study. PLoS ONE. 2019;14(7): e0219963.
8. Ahmed S, Li Q, Liu L, Tsui AO. Maternal deaths averted by contraceptive use: an analysis of 172 countries. The Lancet. 2012;380(9837):111–25.
9. Darroch JE, Sully E, Biddlecom A. Adding it up: investing in contraception and maternal and newborn health, 2017—supplementary tables. New York: The Guttmacher Institute; 2017.
10. Zegeye B, Ahinkorah BO, Idriss-Wheeler D, Olorunsaiye CZ, Adjei NK, Yaya S. Modern contraceptive utilization and its associated factors among married women in Senegal: a multilevel analysis. BMC Public Health. 2021;21(1):1–3.
11. Dadi D, Bogale D, Minda Z, Megersa S. Decision-making power of married women on family planning use and associated factors in Dinsho Woreda, South East Ethiopia. Open Access J Contracept. 2020;11:15.
12. Ahinkorah BO, Hagan JE Jr, Seidu AA, Sambah F, Adoboi F, Schack T, Budu E. Female adolescents' reproductive health decision-making capacity and contraceptive use in sub-Saharan Africa: What does the future hold? PLoS ONE. 2020;15(7): e0235601.
13. Appiah F, Seidu AA, Ahinkorah BO, Baatiema L, Ameyaw EK. Trends and determinants of contraceptive use among female adolescents in Ghana: analysis of 2003–2014 demographic and health surveys. SSM-population health. 2020;1(10): 100554.
14. Ahinkorah BO. Predictors of modern contraceptive use among adolescent girls and young women in sub-Saharan Africa: a mixed effects multilevel analysis of data from 29 demographic and health surveys. Contraception and reproductive medicine. 2020;5(1):1–2.
15. Singh S, Darroch JE. Adding it up: costs and benefits of contraceptive services—estimates for 2012. New York: Guttmacher Institute and United Nations Population Fund (UNFPA). 2012. http://www.guttmacher.org/pubs/AIU-2012-estimates.pdf.
16. Sidibe AM, Kadetz PI, Hesketh T. Factors impacting family planning use in Mali and Senegal. Int J Environ Res Public Health. 2020;17(12):4399.
17. Barden-O'Fallon J, Traore A, Tounkara M. Attitudes and opinions of young people in Mali on family planning, barriers to contraceptive use, and suggestions for programming. Afr J Reprod Health. 2020;24(2):106–14.
18. Kaneda T, Dupuis G. World population data sheet with a special focus on youth. Washington: Population Reference Bureau; 2017.
19. Maiga M, Aissatou L. Repositioning family planning in Senegal: a baseline. Washington: Futures Group; 2012. p. 1–6.
20. Hernandez JH, Muanda M, Garcia M, Matawa G. Awareness and perceptions of emergency contraceptive pills among women in Kinshasa, Democratic Republic of the Congo. Int Perspect Sex Reprod Health. 2017;43(3):121–30.
21. Ansong JK, Otupiri E, Apenkwa J, Aboagye PK. Women's empowerment and contraceptive use: a community-based survey in peri-urban Kumasi, Ghana. Gates Open Res. 2019;3(1512):1512.
22. Frost AE, Dodoo FN. "The Man Comes to Marry the Woman": exploring adolescent boys' gendered expectations for bridewealth and marriage among the Akwapim of Southern Ghana. Marriage Fam Rev. 2010;46(1–2):41–59.
23. OlaOlorun FM, Hindin MJ. Having a say matters: influence of decision-making power on contraceptive use among Nigerian women ages 35–49 years. PLoS ONE. 2014;9(6): e98702.
24. Uddin J, Pulok MH, Sabah M. Couple's reports of household decision-making, unmet need for contraception, and unintended pregnancy in Bangladesh. Munich Personal RepEc Archive. 2015. https://mpra.ub.unimuenchenn.de/id/eprint/63757.
25. Story WT, Burgard SA. Couples' reports of household decision-making and the utilization of maternal health services in Bangladesh. Soc Sci Med. 2012;75(12):2403–11.
26. Alemayehu M, Hailesellasie K, Biruh G, Gebrezgabiher G, Tinsae F, Kidanemariam A, Brhane Y. Married women's autonomy and associated factors on modern contraceptive use in Adwa Town, Northern Ethiopia. Science. 2014;2(4):297–304.
27. Eshete A, Adissu Y. Women's joint decision on contraceptive use in Gedeo zone, Southern Ethiopia: a community based comparative cross-sectional study. Int J Family Med. 2017;2017. https://doi.org/10.1155/2017/9389072.
28. Fuseini K, Kalule-Sabiti I, Lwanga C. Dynamics of women's autonomy in household decision-making in Ghana. J Comp Fam Stud. 2019;50(4):293–312.
29. Tadesse M, Teklie H, Yazew G, Gebreselassie T. Women's empowerment as a determinant of contraceptive use in Ethiopia further analysis of the 2011 Ethiopia demographic and health survey. DHS Further Anal Rep. 2013;82. Calverton, Maryland, USA: ICF International.
30. Do M, Kurimoto N. Women's empowerment and choice of contraceptive methods in selected African countries. Int Perspect Sex Reprod Health. 2012;1:23–33.
31. Corsi DJ, Neuman M, Finlay JE, Subramanian SV. Demographic and health surveys: a profile. Int J Epidemiol. 2012;41(6):1602–13.
32. Von Elm E, Altman DG, Egger M, Pocock SJ, Gøtzsche PC, Vandenbroucke JP, Strobe Initiative. The strengthening the reporting of observational studies in epidemiology (STROBE) statement: guidelines for reporting observational studies. Int J Surg. 2014;12(12):1495–9.
33. Lai SL, Tey NP. Contraceptive use in Cambodia: does household decision-making power matter? Cult Health Sex. 2020;22(7):778–93.
34. Ahinkorah BO, Seidu AA, Appiah F, Budu E, Adu C, Aderoju YB, Adoboi F, Ajayi AI. Individual and community-level factors associated with modern contraceptive use among adolescent girls and young women in Mali: a mixed effects multilevel analysis of the 2018 Mali demographic and health survey. Contracept Reprod Med. 2020;5(1):1–2.
35. Carlile L. An outline of contraceptive use in Mali. Independent Study Project (ISP) Collection. 1255. https://digitalcollections.sit.edu/isp_collection/1255.
36. Blackstone SR, Nwaozuru U, Iwelunmor J. Factors influencing contraceptive use in sub-Saharan Africa: a systematic review. Int Q Community Health Educ. 2017;37(2):79–91.
37. Uddin J, Hossin MZ, Pulok MH. Couple's concordance and discordance in household decision-making and married women's use of modern contraceptives in Bangladesh. BMC Womens Health. 2017;17(1):1.

Publisher's Note

Springer Nature remains neutral with regard to jurisdictional claims in published maps and institutional affiliations.
